# Advances in Robotic Transaxillary Thyroidectomy in Europe

**DOI:** 10.1007/s40137-017-0180-7

**Published:** 2017-06-26

**Authors:** Micaela Piccoli, Barbara Mullineris, Daniele Santi, Davide Gozzo

**Affiliations:** 1 Division of General, Emergency Surgery and New Technologies, OCSAE (Ospedale Civile Sant’Agostino Estense), Via Giardini 1455, Baggiovara, Modena, Italy; 2 Unit of Endocrinology, Department of Biomedical, Metabolic and Neural Sciences, University of Modena and Reggio Emilia, Modena, Italy

**Keywords:** Robotic surgery, Thyroidectomy, Robot-assisted surgery

## Abstract

**Purpose of review:**

The robotic surgical approach for minimally invasive thyroid surgery has been well described from the Korean surgeons and shows a wide spread diffusion in Asian area. This paper gives a systematic review aiming to pointed out the interest and the way of behaving of the European surgeons about the role of the robotic thyroidectomy (RT).

**Recent findings:**

A literature search was performed using Pubmed, MEDLINE, Cochrane and ClinicalTrials.gov databases, including only papers wrote from european surgeons enrolling patients operated in Europe. Outcomes of interest included patients characteristics, patients position, surgical devices, surgical technique, surgical outcomes, and complications. Eighteen studies have been included in the analysis, published from 2011 to 2017. An overall number of 1108 patients were treated in studies included. In the 44.4% of studies (eight trials), the Kuppersmith position was chosen, whereas in the 22.2% (four trials), the Chung position was selected, with a mean length on axilla skin incision of 5.8 ± 1.5 cm. Considering the characteristics of the surgical technique, the mean total surgical time was 166.8 ± 36.6 min (including total thyroidectomy and loboisthmectomy together), divided three consecutive phases, such as mean working space was 50.7 ± 21.8 min, mean docking time 16.0 ± 11.9 min and mean console time 102.87 ± 38.8 min. Considering the complications, only 50% of studies included reported data about acute complications. In particular, the most frequent was hypocalcemia, occurring in 32 cases (2.9%). RLN palsy occurred in 29 patients (2.6%), definitive in 13.8% of these cases and transient in 86.2%. Only nine studies reported the discharge time, with a mean of 2.4 ± 1.2 days after surgery.

**Summary:**

Despite the papers included in the study show a different way of collecting data, the transaxillary approach for robotic thyroidectomy for European patients is both feasible and safe. This procedure has to be carried out by surgeons expert in thyroid surgery with knowledge in robotic procedure. In the future, the incoming of dedicated instruments could improve and developed this technique.

## Introduction

The robotic technology occupies a wide space in surgical complex procedure [1–4], in particular the da Vinci System (Intuitive Surgical, Mountain View,CA). Surgeons all over the worlds well knows the advantages of performing a surgical procedure using robot: instruments that increased precision and avoid tremor transmission, magnification of the operative field, three-dimensional vision and high definition and not least the surgeon comfort. Since Kang et al. first described the robotic transaxillary approach for thyroid (RATS) [5]. Successively, a considerable number of surgeons starts to think that an extracervical approach to thyroid, with a scarless neck procedure, could be easy with the use of the robot instead of the endoscopic methods. Also surgeons that are not used to perform thyroidectomy with and endoscopic extracervical approach were fascinated by the use of the robot. Although this technique has been extensively applied in Asia, the number of patients who undergo RATS is still limited in Europe and USA. One of the most common causes is the anthropometric and weight status-related differences that exist between the Korean and Caucasian patients [6, 7]. Our study carried out a systematic review to the current status for RATS procedures and outcomes in Europe.

## Methods

A systematic search of the literature was performed, using four different databases, such as PubMed, MEDLINE, Cochrane and ClinicalTrials.gov up to April 2017. The following mesh and keywords were included: “robotic thyroidectomy,” “robot-assisted thyroidectomy,” “robot-assisted thyroid surgery.” English language was a restriction. The inclusion criteria were as follows: (i) studies reporting at least one outcome of interest; (ii) studies focusing on patients operated in Europe. Exclusion criteria were: (i) papers written by European surgeon but enrolling patients operated in other countries and by other surgeons; (ii) case report, expert opinion. All data were extracted from a standard form according to protocol by only one reviewer.

For each study included in the review, the following characteristics were considered: (i) characteristics of the paper, such as authors, year of publication, country and number of patients enrolled, (ii) characteristics of patients, such as age, sex, mean size tumor, initial pathology and body mass index (BMI), (iii) characteristics of surgical technique, such as extent of thyroidectomy, patient position, nerve monitoring, length axilla skin incision, retractor type and type of surgical approach of robotic thyroidectomy), (iv) surgical outcomes, such as operation time, length of hospital stay, conversion to cervical anterior approach, transient hypocalcemia, permanently and transient recurrent laryngeal nerve (RNL) palsy, hematoma, postoperative bleeding seroma, wound suppuration, subcutaneous tunnel infection, burn skin, discomfort, internal jugular vein lesion, external jugular vein lesion, tracheal membrane perforation, wound suppuration, discomfort, and dysphagia.

## Results

Eighteen studies have been included in the analysis [6–8, 9••, 10–23], published from 2011 to 2017. Eight were published in French population, four in Italy, two in Germany, two in Romania, one in Greece and one in United Kingdom (Table 1). An overall number of 1108 patients were treated in studies included. The age was reported in 11 studies, with a mean age of 43.6 ± 5.7 years. Similarly, BMI was reported in nine studies, with a mean value of 25.3 ± 6.9 kg/m^2^ (Table 1). Only seven studies reported the mean tumor size, with an average value of 2.7 ± 0.4 cm.

**Table 1:** Characteristics of patients included in studies evaluated

Author	Year	Country	Number of patients (n)	Mean age (Years)	Gender (n F/M)	Mean tumor size (cm)	Mean BMI (kg/m^2^)	Disease
Lallemant et al.	2011	France	21	53.5	18/3	<5	NM	B/FA/PC
Ciabatti	2012	Italy	29	45.0	24/5	<6.5	<35	PC
Kiriakopoulos	2012	Greece	8	38.8	NM	2.6	23.4	DT3; 5B
Axente et al.	2013	Romania	50	47.5	49/1	3.2	43.2	50B;
Boccara et al.	2013	France	20	49.8	17/3	NM	23.9	NM
Lallemant et al.	2013	France	23	42.6	21/2	2.9	24.4	18FA/1B/4 graves + B
Aidan et al.	2013	France	46	43.2	44/2	NM	21.8	B/DT
Materazzi et al.	2014	Italy	32	32.5	31/1	1.83	20.9	19B; 10FA; 3TA
Rabinovics et al.	2014	France	190	NM	NM	NM	NM	B
Abramovic et al.	2015	France	26	NM	NM	<5	low	B
Al Kadah & Piccoli et al.	2015	Germany/Italy	16	Range 17–55	5/11	NM	NM	14B; 2 IHPT
Espiard et al.	2015	France	60	NM	NM	NM	NM	NM
Lorincz et al.	2015	German	10	NM	6/4	<4	<30	NM
Piccoli et al.	2015	Italy	196	NM	NM	2.9	NM	5IHPT; 120B; 38FA; 38PC
Rabinovics et al.	2015	France	212	45.0	185/27	>20 ml	23.0	NM
Arora et al.	2016	UK	16	42.0	16/1	3.0	25.9	16B
Axente et al.	2016	Romania	91	NM	88/3	NM	>25 and <30	
Fregoli et al.	2017	Italy	62	39.7	NM	2.6	20.9	21B; 19FA; 12PC

*B* benign;* DT* docking time;* FA* follicular adenoma;* IHPT* Primary Hyperparathyroidism;* NM* not mentioned;* PC* papillary carcinoma(PT1a);* TA* toxic adenoma;* TDT* thyroid differentiated tumor

The extent of thyroidectomy was reported in 14 studies (77.8%) (Table 2). In the 44.4% of studies (eight trials), the Kuppersmith position was chosen, whereas in the 22.2% (four trials), the Chung position was selected, with a mean length on axilla skin incision of 5.8 ± 1.5 cm (Table 2). Only two studies over 18 provided the intermitted nerve monitoring. Considering the characteristics of the surgical technique, the mean total surgical time was 166.8 ± 36.6 min, divided three consecutive phases, such as mean working space was 50.7 ± 21.8 min, mean docking time 16.0 ± 11.9 min, and mean console time 102.87 ± 38.8 min (Table 2).

**Table 2:** Characteristics of surgical technique

Author	Extent of thyroidectomy	Patients position	Nerve monitoring	Length axilla skin incision (cm)	Retractor type	Working space	Mean working space (min)	Mean docking time (min)	Mean console time (min)	Mean total time (min)	RT Surgical approach
Lallemant et al.	TT16	NM	NM	NM	NM	NM	51	NM	83	197	ICA
Ciabatti	TT29	CP	NM	8–9	CR	DV	19.7	11.9	126.10	178.51	TAA
Kiriakopoulos	HT3; PT2; TT3; CCND1	NM	NM	4–6	CR	DV	32	13	166	211	TAA
Axente et al.	HT33; PT8; 9TT	CP	NM	4–5	CR	DV	70.3	9.6	68	159	TAA
Boccara et al.	HT14; TT4; TPT2	KP	INM	NM	KR	NM	NM	NM	NM	185	TAA
Lallemant et al.	HT14; TT9	KP	NM	6–8	CR	DV	49	NM	66	134	TAA
Aidan et al.	HT31; PT3; TT13	6CP; 40KP	NM	5–6	CR22; 25 KR	DV	NM	NM	NM	NM	TAA
Materazzi et al.	HT32	NM	NM	5–7	CR	NM	NM	9.4	NM	84.25	TAA
Rabinovics et all	TT98; PT82; TPT10; CCND 17	NM	NM	NM	NM	DV	NM	NM	NM	142PT 170TT	TAA
Abramovic et al.	NM	KP	INM	6–9	CR	DV	15	NM	105	175	TAA/DVSi
Al Kadah Piccoli et al.	HT12; PT2; PTx2	KP	NM	5	MR8 TLR8	FHL8 EC8	60	15	NM	NM	TAA
Espiard et al.	NM	NM	NM	NM	NM	NM	NM	NM	NM	NM	TAA
Lorincz et al.	HT6; TT4	KP	NM	5–6	MR	EV	77	45	159	NM	TAA
Piccoli et al.	NM	KP	NM	4–5	MR	EV	63.8	14.9	39.9LT 77.1TT	160.2TT 115.1LT	TAA
Rabinovics et all	TT 110; PT90; TPT12; CCND 17	KP	NM	5–6	NM	DV	NM	NM	NM	140 PT 170 TT	TAA
Arora et al.	HT16	CP	NM	6.1	NM	DV	NM	NM	NM	228	TAA
Axente et al.	HT50; TT22PT19;	CP	NM	5	CR	CV	69.35	9.19	75.65	164.26	TAA
Fregoli et al.	NM	NM	NM	5-7	CR	NM	NM	NM	NM	119.4	TAA

*CCND* central compartment neck dissection;* CP* Chang position;* CR* Chung retractor;* DV* direct vision;* DVSi* Da Vinci Si;* EC* endoscopic camera;* FHL* frontal head light;* HT* hemithyroidectomy;* ICA* infraclavicular approach;* INM* intermitted nerve monitoring;* KP* Kuppersmith position;* KR* Kuppersmith retractor;* MR* Modena Retractor;* NM* not mentioned;*PT* partial thyroidectomy;* PTx * parathyroidectomy; *RT* robotic thyroidectomy;* TAA * transaxillary access;*TLR* Tuttlingen;* TPT* target parathyroidectomy;* TT* totalthyroidectomy

Only in 14 over 1108 patients (1.3%), the surgical conversion was performed and in six patients (0.5%) a surgical revision was performed (Table 3). Considering the complications, only 50% of studies included reported data about acute complications (Table 3). In particular, the most frequent was hypocalcemia, occurring in 32 cases (2.9%) (Table 3). Among the others acute complications, hematoma occurred in 10 patients (0.9%) (Table 3). RLN palsy occurred in 29 patients (2.6%), definitive in 13.8% of these cases and transient in 86.2%. Only nine studies reported the discharge time, with a mean of 2.4 ± 1.2 days after surgery (Table 3).

**Table 3:** Complications

Study	year	Conversion (n)	Revision surgery	Postop. bleeding In 48 h (cl)	Acute complications	RLN palsy	Brachial plexus	Dysesthesia	Discharged days
Lallemant et al.	2011	2	0	NM	NM	1 T; 2 P	0	0	NM
Ciabatti	2012	0	NM	NM	NM	2 T	NM	NM	NM
Kiriakopoulos	2012	0	0	NM	NM	1 T	0	3 D	1.5
Axente et al.	2013	1	NM	1	1HC; 3S; 1WS	1 T	1 T	NM	4.3
Boccara et al.	2013	0	0	97.7	1HE	0	0	0	Max 3
Aidan et al.	2013	1	0	NM	1HE; 1D	4 T; 1 P	2 T	38 D	3.2
Lallemant et al.	2013	1	0	NM	2HE; 1IGV	0	0	11 D	1
Materazzi et al.	2014	NM	NM	NM	NM	NM	NM	NM	1.8
Rabinovics et al.	2014	4	2	NM	NM	0	8 T	0	NM
Abramovic et al.	2015	1	0	NM	NM	6 T	0	26 D	2
Al Kadah Piccoli et al.	2015	NM	NM	NM	NM	NM	NM	16 DS 2 D	NM
Espiard et al.	2015	0	0	NM	NM	0	0	0	NM
Lorincz et al.	2015	0	0	NM	1IGV; 1TMP	1 T	0	0	NM
Piccoli et al.	2015	NM	NM	NM	2BS; 27HC; 1IGVL; 1EGVL; 4S; 4HE; 1STI	7 T	6 T	NM	NM
Rabinovics et al.	2015	4	4	NM	NM	1 P	9 T	0	No difference with conventional
Arora et al.	2016	NM	NM	NM	1S	1 T	1 T	NM	1
Axente et al.	2016	NM	NM	NM	1S; 2HC; 1HE; 1WS	1 T	NM	5 D	NM
Fregoli et al.	2017	0	NM	1	3HC; 1HE	NM	NM	NM	3.9

*BS* burn skin;* D* dysphagia;* DS* discomfort;* EGVL* external jugular vein lesion;* HC* Hypocalcemia;* HE* hematoma;* IGVL* Internal jugular vein lesion;* P* Permanent;* RLNS * seroma;* STI * subcutaneous tunnel infection;* T* Transient;* TMP* tracheal membrane perforation;* WS* wound suppuration

## Discussion

This research carried out a systematic review of the literature published from 2011 to 2017, aiming at identifying the use of RATS only in Europe. The South Korean surgeons published the first paper about transaxillary gasless thyroidectomy, and they suggest surgical indication, outcomes, type of instruments, associated technologies, costs, concluding that this technique is feasible and can be safely performed in selected population [7]. Also some of the American surgeons introduced RATS procedure in their practise but after few cases, performed by expert surgeon, they conclude that the main benefit of this procedure (i.e., the translocation of the surgical skin incision to the axilla) did not offset the risk and liability of performing this kind of operation. Moreover, they add that they could perform RATS but not that they should [24]; but Berber [25••], four years later, suggest that robotic remote-access thyroidectomy may be done safely in high volume centers.

Analyzing the Korean papers about robotic transaxillary thyroidectomy, it is possible to see an homogeneous way in describing the characteristics of the patients, the surgical technique and the outcomes [5]. This feature is possible because all the literature relies on the singular experience of a group of South Korean surgeons, working in different institutions, but with the same medical background and in the same country. On the contrary, the evaluation of European dataset is still challenging. In particular, the majority of European patients treated with these procedure are female with a mean BMI value of 25.3 ± 6.9 kg/m^2^. Axente et al. correlated the incidence of complication and postoperative evolution in 3 different BMI groups (BMI < 25; 25 < BMI < 30; BMI > 30) and concluded that there were no significant differences between BMI groups and the procedure was considered equally safe irrespective of the presence or absence of obesity [7]. The most common disease treated by RATS were benign thyroid lesions, whereas few centers treated malignant tumors, and only one study reported central lymph node dissection [22] and none described lateral neck dissection. This first result demonstrates that we need more data to assess the oncological validity. Target parathyroidectomy alone or associated with thyroidectomy is described [7, 9••, 10, 12–21, 23].

**Fig. 1:**
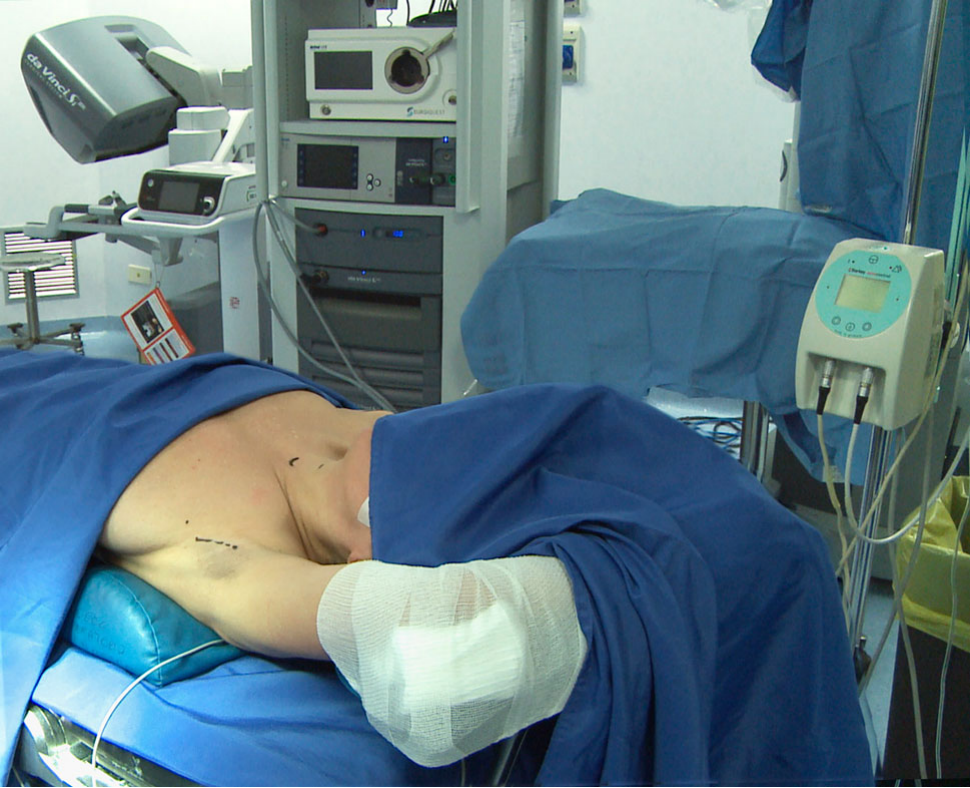
Patient positioning

**Fig. 2:**
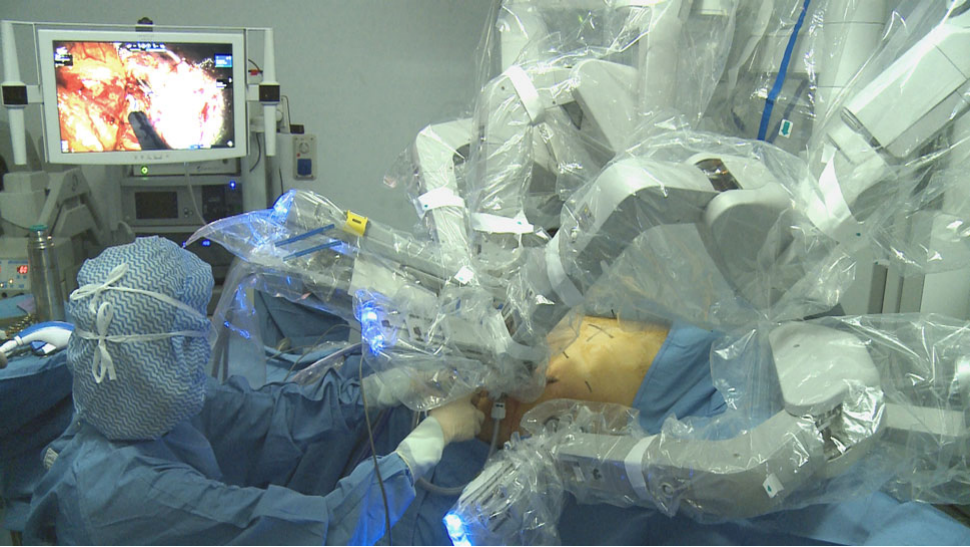
Docking is shown

**Fig. 3:**
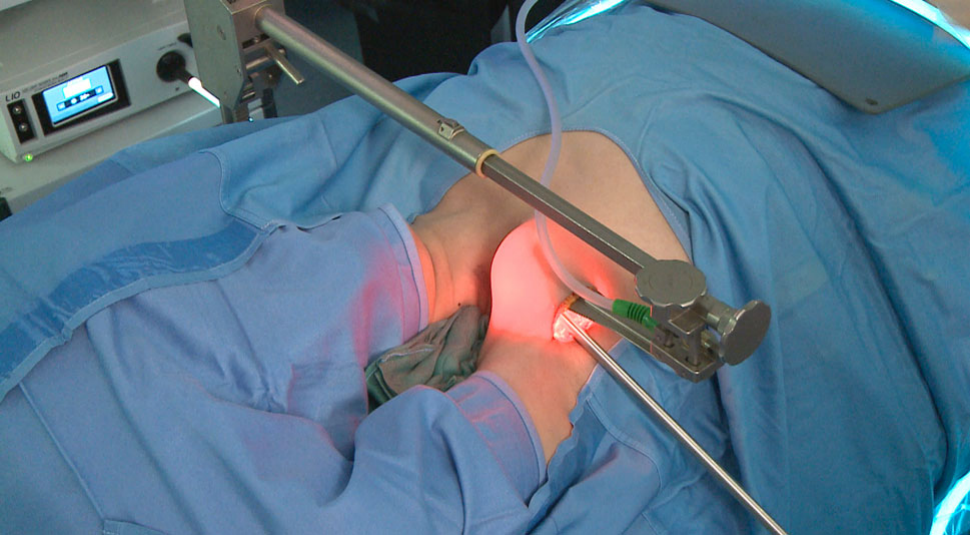
The Modena retractor and endoscopic vision for the flap

The position of the patient, more than the cervical approach, is very import to avoid specific complication not usually seen in the cervical thyroid method. The first position described is the patient placed supine under general anesthesia, the neck slightly extended, and the lesion-side arm raised and fixed to make shortest distance from the axilla-Chung position (CP) [7]. The second position is a modified arm positioning before general anesthesia to avoid brachial plexus neurapraxia: forearm is bent at 90° and arm position is checked in the operative room—Kuppersmith position (KP) [26]. The Korean papers reported only the first position and compare the outcomes, whereas the European surgeons wrote papers using the two positions. Thus, it is very difficult to compare the outcomes of different surgical approaches. Conversion to an open neck approach is described by different authors in both position [6, 8, 11, 17, 21], but the major number of conversion has been described in patient in KP, so we can not assume that the CP for European patients is worst than the KP. The length of the axilla skin incision with a mean length of 5.8 ± 1.5 cm depends on the technical devices used to perform the flap to reach the thyroid. A shorter incision has been described by Piccoli [23] with the use of endoscopic vision during the creation of the working space (Figs. 1, 2, 3). The Korean surgeons use only a direct vision to perform the working space with a 5 to 6 cm vertical skin incision [7]. Also different external retractor are used in western countries: Modena retractor (MR), Chung retractor (CR), and Kuppersmith retractor (KP) with the difference that the MR can be used from the beginning of the operation and can be handled by only one surgeon at the operating table avoid the effort of two surgeons lifting up the flap [23]. Shoulder discomfort, dysesthesia, brachial plexus, and internal/external jugular vein injury are difficult to compare if we use different external retractor instead of only the CR as happen in Korea.

The RATS is a surgical multistep technique dived in consecutive phases, such as working space, docking time and console time [7, 11–15]. From this review not all the step are described, sometimes the working space and the docking time are considered together, other author in the total operative time do not consider the docking [20], so that it is difficult to know which is the step more time-consuming and technically demanding. The outcomes results comparable to other conventional technique in terms of postoperative hypocalcemia, recurrent laryngeal nerve palsy, definitive laryngeal nerve, and hospital length stay [27•]. Only one paper reported a tracheal membrane perforation [19] due to the necessity to overcome the learning curve for a procedure that require expert surgeon in thyroid and robotic procedure.

The anesthetic implication for RATS include all the steps, beginning from the position of the ipsilateral upper limb, avoiding brachial plexus injury; anesthetic monitoring and management of the patients during a prolonged surgery; anticipation of postoperative analgesia [12]. Scar satisfaction is a clinical data not always reported. This data is very important if we consider that RATS is performed to avoid neck scar. The satisfaction is not related to the result of the scar but from the scar neck distance. Lallemant [17] described the cosmetic results of the scar and 16 patients on a total of 20 were either satisfied or very satisfied. Materazzi [20] with a patient scar assessment questionnaire compare two different thyroid techniques: the RATS and the minimally invasive video-assisted thyroidectomy (MIVAT). The appearance and satisfaction with scar appearance scores significantly favored MIVAT. The author concluded that it might be the length of the scar even if it is hidden in the axilla. All the papers, except one, describe the transaxillary robotic approach to remove the thyroid gland. Lallemant [18] at the beginning of his robotic thyroid experience describe an infraclavicular approach. Due to the technical difficulties, he concluded that this technique is feasible, but not safe enough. Among the eighteen papers, only two compare the RATS to another technique: MIVAT versus RATS [19] and conventional cervical approach versus RATS [10]. They both analyzed the cosmetic results and Arora added also postoperative pain, recurrent laryngeal nerve injury, and seroma. A relative small number of patients were recruited in both studies but they can assert that the transaxillary procedure is safe and feasible in selected patients.

Finally, the number of robotic arms and the position used to perform a total thyroidectomy is not the same in all papers, in particular Fregoli [15] used three arms and Piccoli [23] used four arms.

## Conclusion

The papers included in the study have collected data heterogeneously, had different end points, and therefore present difficulties for a comparison. A major reason is that the surgeons have different backgrounds and that the techniques are new and evolving. Likewise, we have not standardized the procedure yet. With the differences in technique, it is not possible to compare the European with the Korean experience.

We suggest at least to divide the robotic transaxillary thyroidectomy in three steps: working space, docking time, and console time. For each step it is necessary to describe the time spent and the technical devices used, the patient position, in order to analyze which one has less complications, and all major postoperative complications, in order to have more data to compare and to identify opportunities to refine the technique. The literature so far though suggests that the transaxillary approach for robotic thyroidectomy is both feasible and safe. However, this procedure needs to be carried out by surgeons with expertise in thyroid surgery and robotic technology. In the future, the incoming of dedicated instruments could improve and develop this technique further.
